# Lithocholic bile acid induces apoptosis in human nephroblastoma cells: a non-selective treatment option

**DOI:** 10.1038/s41598-020-77436-w

**Published:** 2020-11-23

**Authors:** Julian Trah, Jonas Arand, Jun Oh, Laia Pagerols-Raluy, Magdalena Trochimiuk, Birgit Appl, Hannah Heidelbach, Deirdre Vincent, Moin A. Saleem, Konrad Reinshagen, Anne K. Mühlig, Michael Boettcher

**Affiliations:** 1 Department of Pediatric Surgery, University Medical Center Hamburg-Eppendorf, Martinistrasse 52, 20246 Hamburg, Germany; 2 Department of Pediatric Nephrology, University Medical Center Hamburg-Eppendorf, Martinistrasse 52, 20246 Hamburg, Germany; 3 Department of Pediatric Nephrology, University of Bristol, 24 Upper Maudlin St, Bristol, UK

**Keywords:** Paediatric research, Paediatric cancer

## Abstract

Lithocholic bile acid (LCA) has been reported to selectively kill cancer cells within many tumor cell lines including neuroblastoma or glioblastoma. Wilms’ tumor shares similarities with neuro- and glioblastoma. Hence, the aim of the study was to evaluate the effects of LCA on nephroblastoma. To test the effects of LCA, nephroblastoma cell line WT CLS1 was used. SK NEP1 was tested as well. It was originally classified as a nephroblastoma cell line but was meanwhile reclassified as an ewing sarcoma cell line. As control cell lines HEK 293 from embryonic kidney and RC 124 from adult kidney tissue as well as podocytes were used. The effects were evaluated using proliferation assay, caspase activity assay, FACS and Western blot. LCA showed a dose and time-dependent selective effect inducing apoptosis in nephroblastoma cells. However, these effects were not limited to the nephroblastoma cell line but also affected control kidney cell lines and the sarcoma cells; only podocytes are significantly less affected by LCA (at dosages < 200 µm). There were no significant differences regarding the TGR5 receptor expression. The study showed that LCA has a strong, yet unselective effect on all used in vitro cell-lines, sparing the highly differentiated podocytes in lower concentrations. Further studies are needed to verify our results before dismissing LCA as an anti-cancer drug.

## Introduction

Nephroblastoma, also known as Wilms’ tumor is the second most common intraabdominal cancer, and the fifth most common malignoma of children^1^. It is assumed that multiple mutations lead to malignant neoplastic changes in so called nephrogenic rests^2^. More than 95% of all kidney malignancy in the pediatric population are Nephroblastoma^3^. The incidence of the disease reaches its peak at the age of 2–3 years. Intensive research has improved survival rate from less than 30 to 85–90%. However, the relapse rate remains at 15–50%^1,4^.

Therapy and prognosis of nephroblastoma in children is linked to the histological staging^5,6^. The tumor is classified by the Children’s Oncology Group (COG), a surgico-pathologic staging system which is ranking the tumor in 5 groups^1^. Depending on the staging, therapy includes chemotherapy and eventually radiation therapy of the abdomen and in severe cases even radiation of the lungs^5,7^. Chemotherapeutic drugs and radiation therapy lead to long-term effects, especially in very young children^8,9^. The most common long-term side effects is congestive heart failure due to the cardiac toxicity of Doxorubicin which is used in standard chemotherapy protocols for nephroblastoma therapy^5,10^. Other long-term effects are musculoskeletal growth defects, infertility particular in females, and secondary malignancies, which effects approximately 3% of children treated for Wilms’ tumor^8,11,12^. Thus, effective and selective treatment options with a less harmful side effect profile are needed^13^. It has been reported that various bile acids, especially lithocholic bile acid (LCA), have the potential for selective effects on neuroblastoma, breast cancer, androgen-dependent and -independent prostate cancer and glioblastoma^14–16^. Lithocholic bile acid is a secondary bile acid formed by bacterial 7-dehydroxylation of the primary bile acid chenodeoxycholic acid (CDCA) and of the secondary bile acid ursodeoxycholic acid (UDCA)^17–20^.

Like neuroblastoma and glioblastoma, nephroblastoma is an undifferentiated tumor that is caused by malignancy in precursor cells. Hence, the aim of this study was to evaluate the effects of LCA on Wilms’ tumor cell lines.

## Results

### LCA kills cultured human nephroblastoma cells

**Figure 1:**
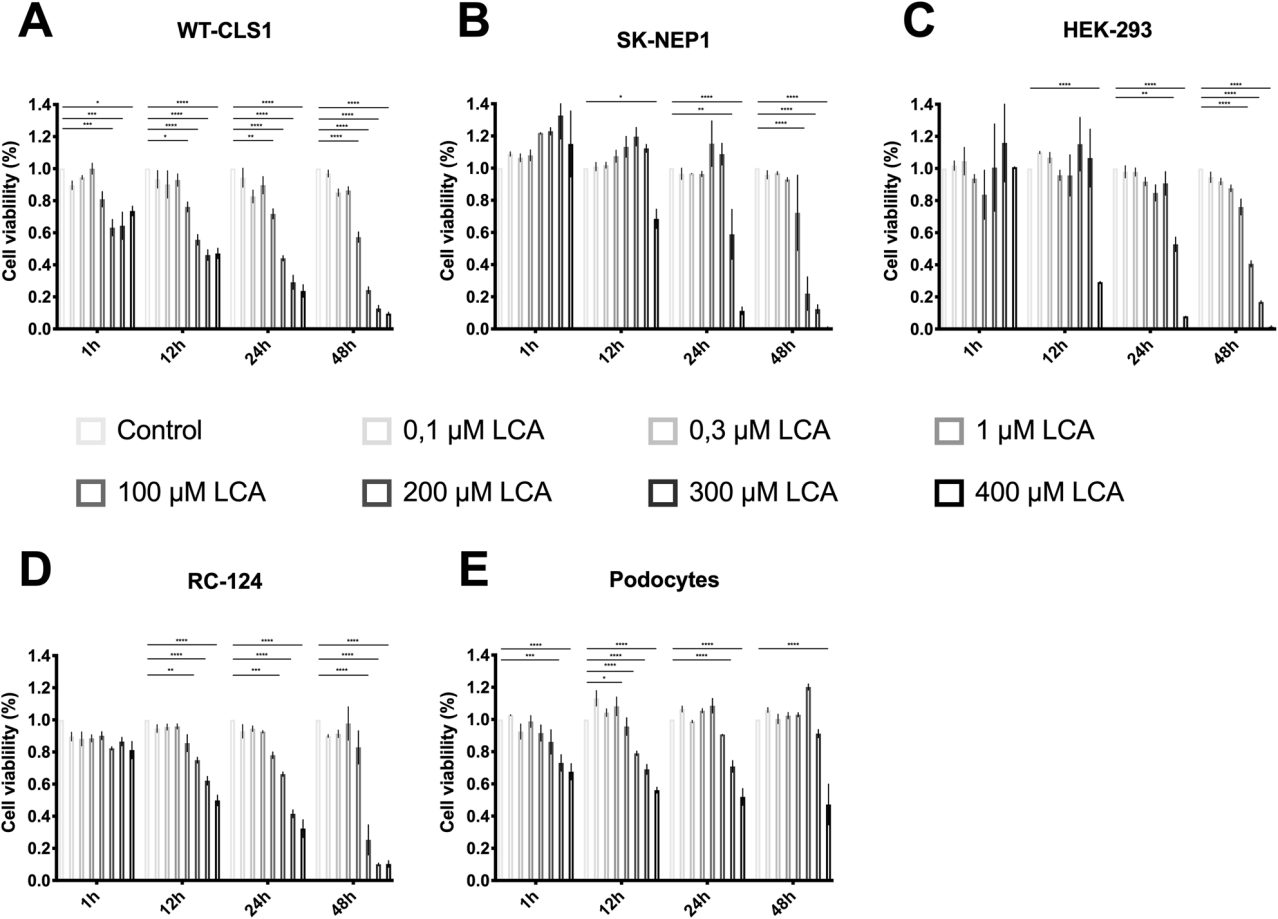
LCA kills cultured human nephroblastoma cells. The percentage of viable cells was calculated using the RealTime-Glo MT Cell Viability Assay**.** All cell-lines were treated as described in “Methods”. Data were evaluated using Prism and are presented as mean ± SD of values from three independent experiments. Significance level was set as *p* < 0.05 (*< 0.05; **< 0.005; ***< 0.0005; ****< 0.0001).

Using the RealTime-Glo MT Cell Viability Assay, LCA showed a potent antiproliferative effect in the used nephroblastoma cell-line and the sarcoma cell-line but also affects the non-tumorous cell-lines HEK 293 and RC 124 as well as the used human podocytes. The cytotoxic effect increases with dosage and time. At 100 µM LCA shows a weak cytotoxic effect on the used cells but the effect is growing in time. Podocytes on contrary show effect for 100 µM and the cell viability is rising with time. With increasing dosage of LCA, the effect is more distinct. In 400 µM LCA the loss of cell viability is severe and the increasing toxicity with time is significantly for all tested cell-lines (Fig. 1).

WT CLS1, SK NEP1, HEK 293 and RC 124 show a dosage and time depending cell toxicity when treated with LCA. Beginning treatment for 4 h, the effect is slightly visible. Only SK NEP1 show a significant stronger toxicity then the other cell-lines. The longer treated, the more toxicity is detected. Treatment for 48 h show the clearest effect in all cell-lines. When treated with 400 µM LCA cell viability of Human Podocytes decreases with time and dosage.

**Figure 2:**
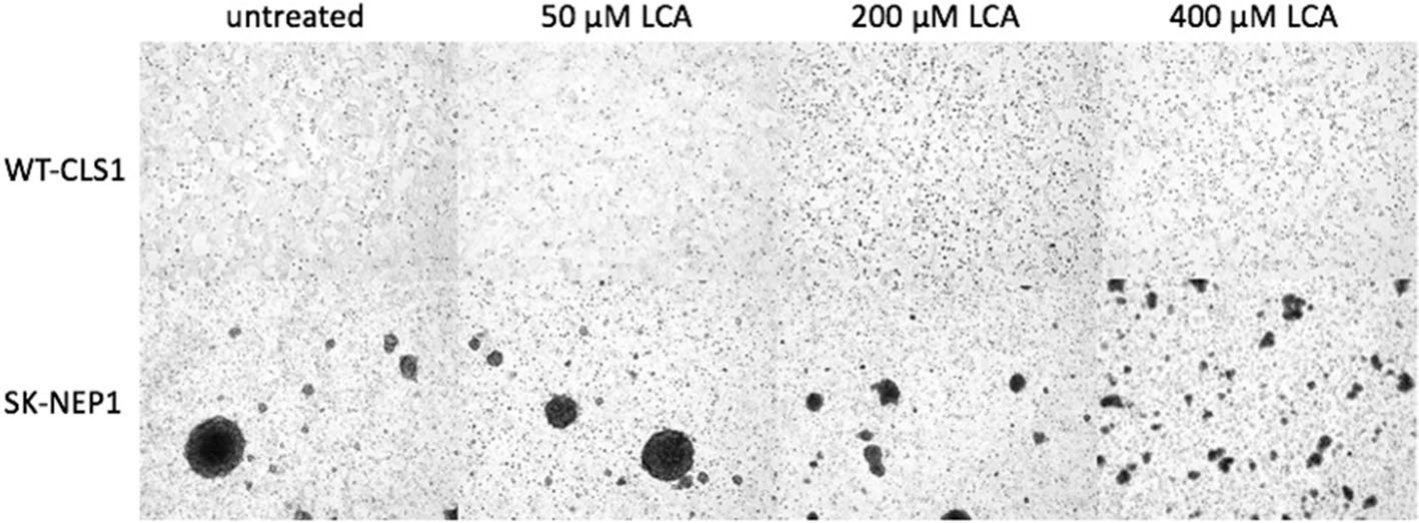
Morphology of the tumor cell-lines WT CLS1 and SK NEP1 and the optical shift due to LCA. WT CLS1 and SK NEP1 were treated with indicated concentrations of LCA for 48 h. Data are representative data of three independent experiments.

Results correlate with the histological appearance of the nephroblastoma cell and the sarcoma cell line as shown in Fig. 2. The untreated adherent cell-lines WT CLS1 grows in a confluent mono-layer while SK NEP1 cells grow spherical. When WT CLS1 is treated for 48 h with increasing concentrations of LCA, the monolayers become leaky. The spheres of SK NEP1 become smaller and show multiple single cells after treatment.

### LCA spares cultured human podocytes

**Figure 3:**
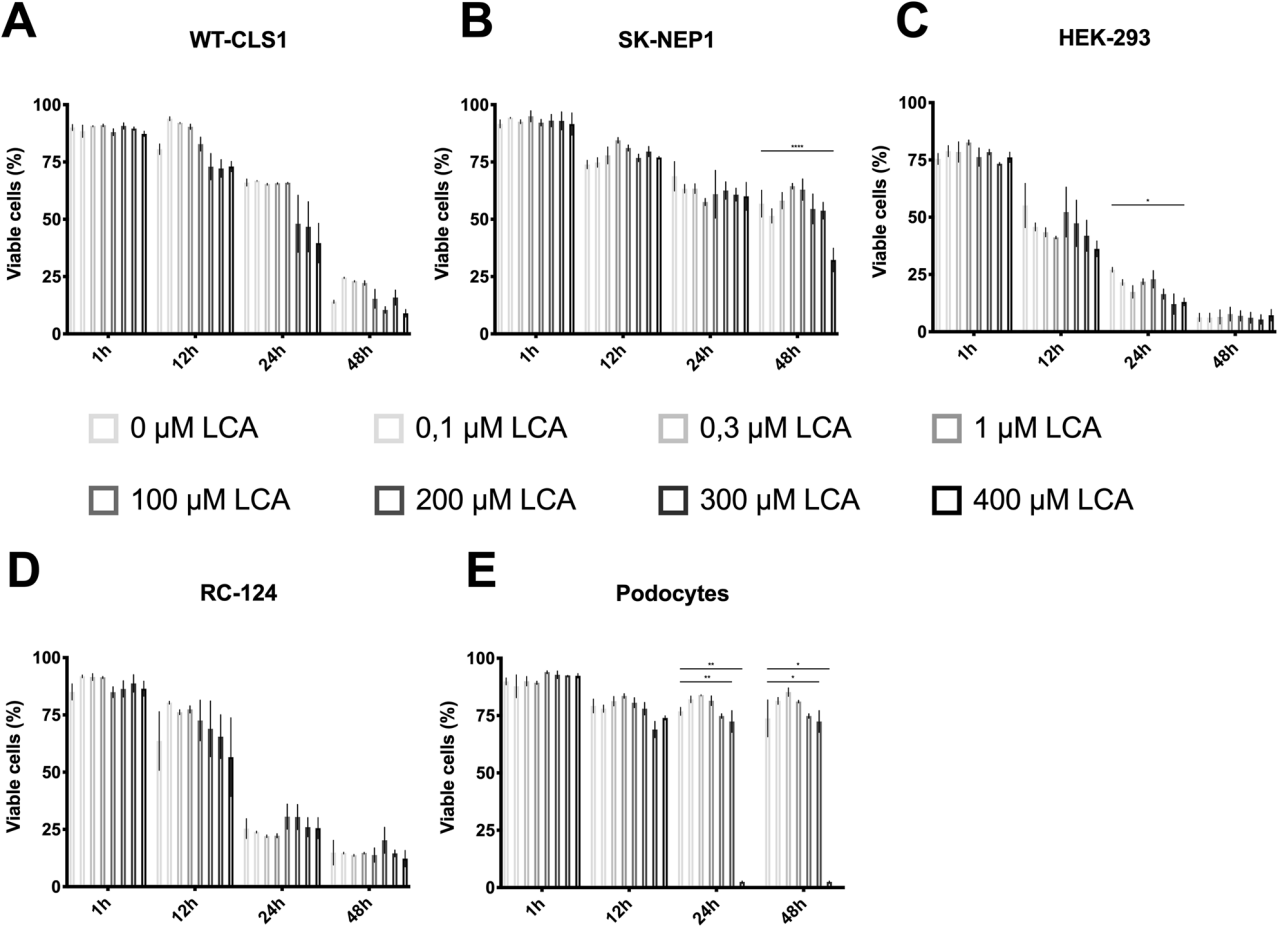
Podocytes versus nephroblastoma cell line under LCA treatment. All cell-lines were treated as described in “Methods”. Cells were stained with Annexin V and PI. Viable cells were defined as Annexin V and PI negative. Data were evaluated using FlowJo and Prism and are presented as mean ± SD of values from three independent experiments. Significance level was set as *p* < 0.05 (*< 0.05; **< 0.005; ***< 0.0005; ****< 0.0001).

FACS analysis were done as seen in Fig. 3, untreated cells showed a high proportion of Annexin V and PI negative cells when treated for 1 h. Similar effects can be seen for all cell-lines in the viability assay. A time dependent as well as a dose depended effect was detected in all cells. SK NEP1 especially show a high percentage of remaining viable cells when treated for 48 h in all concentrations of LCA. Cultured human Podocytes in contrast show a stable level of viability, with the exception of 300 µM and 400 µM LCA at 24 and 48 h only little viable cells left.

### TGR5 Receptor is expressed in all used cell lines without change in NRF2 Expression

**Figure 4:**
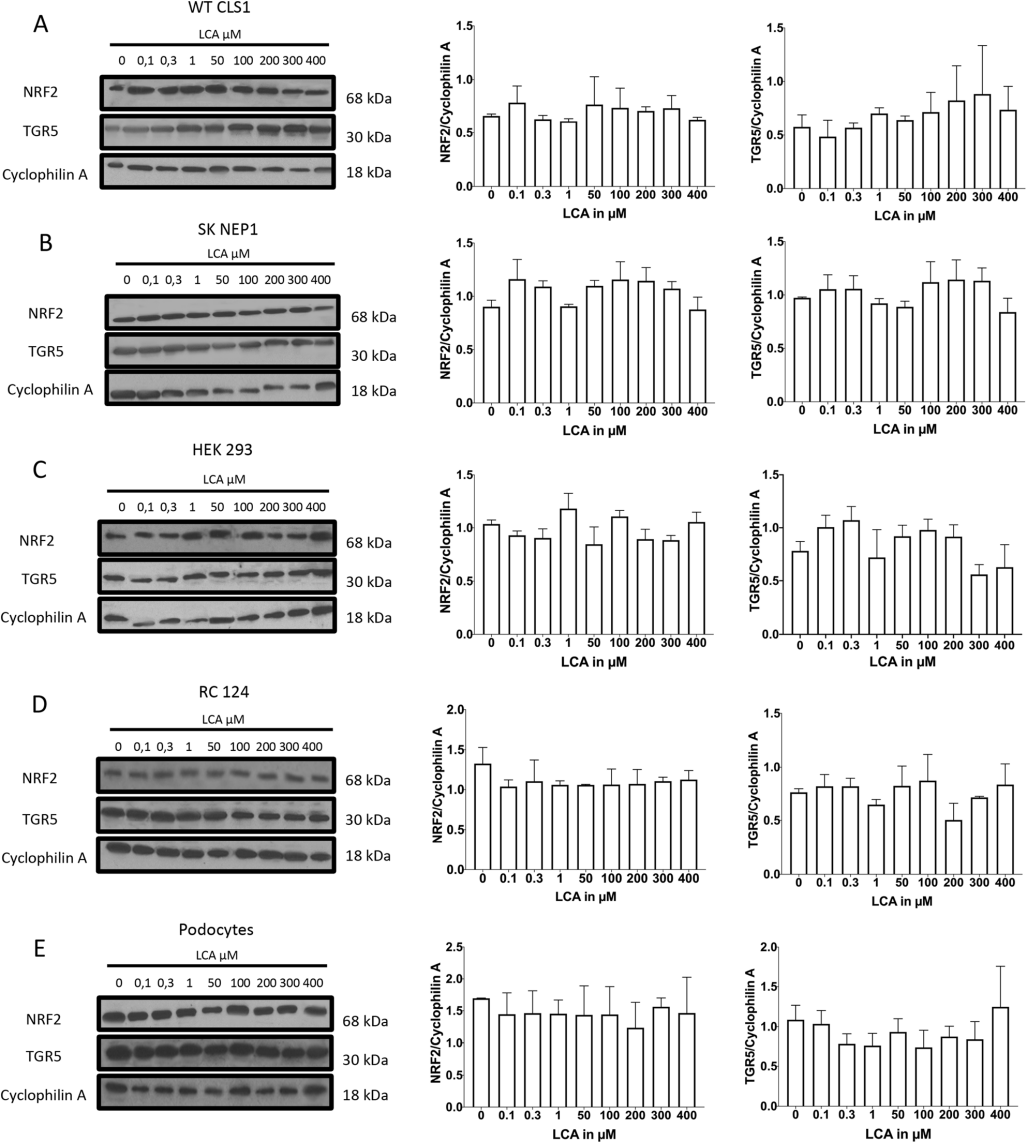
Expression of NRF2 and TGR5. All cell-lines were treated as described in “Methods”. Cell lysates were subjected to immunoblotting with indicated antibodies. Cyclophilin-A served as loading control. Qantification of NRF2 or TGR5 was performed by densitometry. Data were analyzed using ImageStudioLite Version 5.2.5. and ImageJ. Data are presented as mean ± SD of values from three independent experiments. Significance level was set as *p* < 0.05 (*< 0.05; **< 0.005; ***< 0.0005; ****< 0.0001).

TGR5 receptor expression was evaluated using Western blot. It showed expression of TGR5 receptor at 33 kDa in all cell-lines (Fig. 4). Cyclophilin-A was used as housekeeping protein and is visualized at 18 kDa. There is no change in amount of NRF2 at 68 kDa as the downstream regulated protein of the TGR5 pathway (Fig. 4).

### Cell toxicity due to caspase 3 and 7 activation

**Figure 5:**
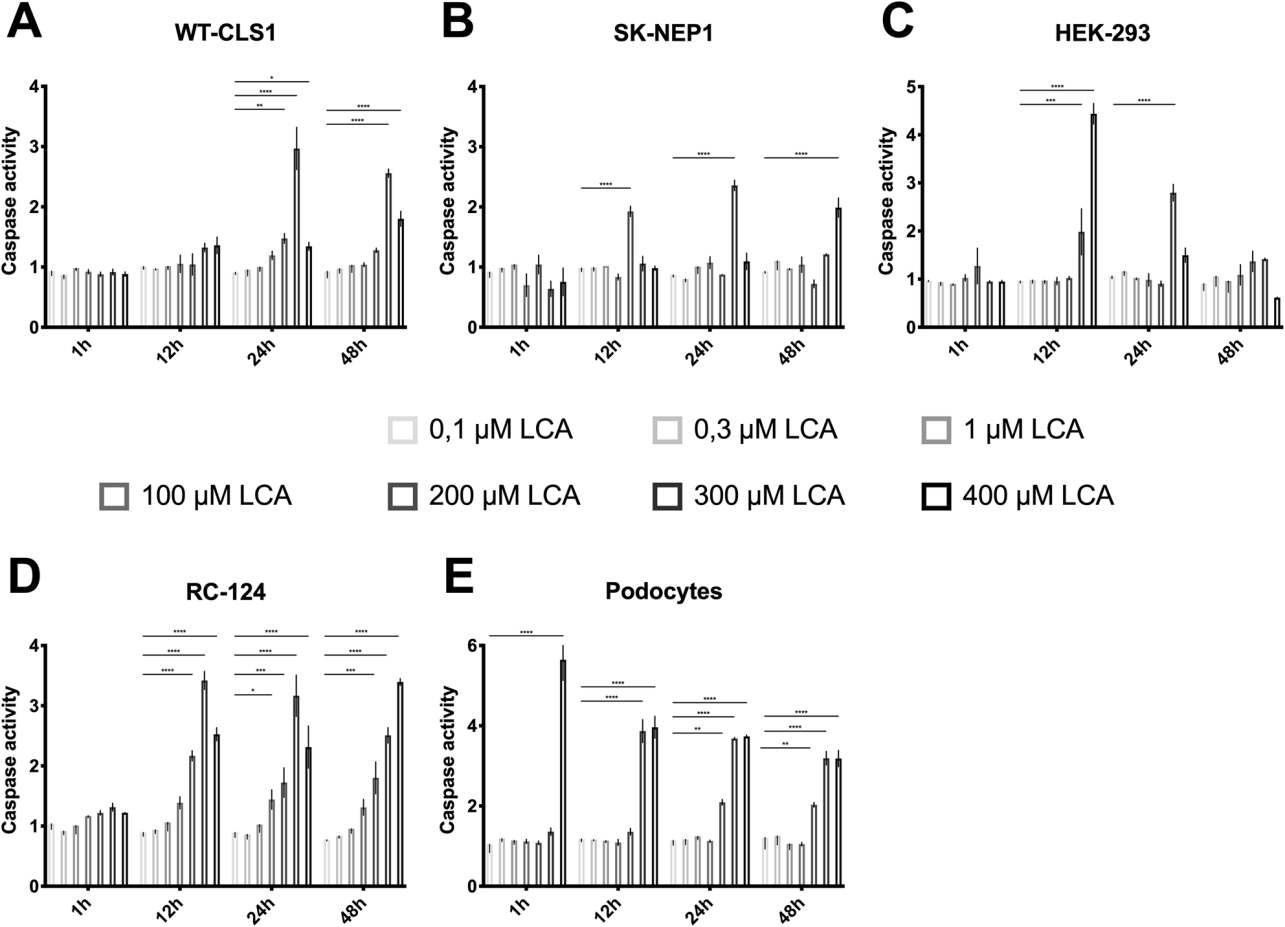
Caspase 3 and 7 activation due to LCA. All cell-lines were treated as described in “Methods” with different doses at different timepoints. Data are presented as mean ± SD of values from three independent experiments. Significance level was set as *p* < 0.05 (*< 0.05; **< 0.005; ***< 0.0005; ****< 0.0001).

The activation of caspase 3 and 7 as modulatory proteins of apoptosis was detected using the Caspase-Glo 3/7 Assay. Treatment with LCA showed a time and dose dependent increasing level of caspase activation (Fig. 5). When treated for 4 h, only cultured human Podocytes show an increasing level of caspase activity that does not correlate with the corresponding assays. When treated for longer, all cell-lines except HEK 293 show an increasing level of caspase activation with time and dosage.

## Discussion

LCA shows a selective dose dependent effect on cultured human nephroblastoma cells whilst slightly affecting the differentiated renal cell-line of podocytes in lower concentrations. This is in line with previous studies that showed LCA selectively kills cultured human tumor cells^14–16^. But unlike these studies, this is the first study that shows that the cytotoxic effect of LCA is not limited to tumor cells like nephroblastoma, neuroblastoma or glioblastoma but might affect all cell-lines. LCA affected nephroblastoma cells like WT CLS1 and sarcoma cells like SK NEP1 as well the control cell lines HEK 293 and RC 124. However, LCA spares human podocytes. As in previous studies examining LCA as anti-cancer treatment, we used highly differentiated cells (human podocytes) as control cells. They were significantly less sensitive to LCA than the tumor cell lines at least in lower concentrations. This would make LCA quite appealing to be used as a treatment of nephroblastoma. However, podocytes are highly differentiated cells with reduced proliferative capacity making them less prone to any unselective cytotoxic treatment^21^. The reference cell-line in a different study examining LCA for neuroblastoma were human neurons cells which are equally differentiated cells^14,16^. The control cell-line for prostate cancer was epithelial prostate cell-line RWPE-1 and shows, as well, a selective effect of LCA^16,22^. Other authors evaluating LCA for the treatment of glioma and breast cancer reported excellent properties of LCA; however, control cell-lines were missing^14^. This study shows that LCA has non-selective cytotoxic effects affecting all fast proliferating cells and postulates that possibly these effects are not limited to tumor cells like glioma, neuro- or nephroblastoma. The underlying mechanism of LCA on tumor cells is still to be solved. Goldberg et al. showed that LCA may not enter the cells, but unfolds its effects on the cell surface^14,16^. Lately Kovács et al. could show that LCA not only exerts its function by binding to the TGR5 Receptor but as well by interaction within the constitutive androstane receptor (CAR) pathway^23^. There are at least 7 LCA receptors known today of whom TGR5 is the most studied in behalf of LCA. The TGR5-receptor is expressed on various types of cells including all cell-lines utilize in this current study^24^. But contrary to the study by Kovács et al., we could not show an influence in the downstream of TGR5 by decreasing of NRF2. Leading to the conclusion that the effects of LCA most likely depend on intracellular mechanisms mediated by TGR5 that are not known so far or regulated by possible other receptors as CAR^14^.

Because of the unselective effects, we doubt that LCA could be utilized as a universal treatment option for tumor cells. In addition, it should be considered that LCA has possible side effects like cholestasis and might contribute to liver injury^17^. The selective effects on cancer cells which have been reported until today may result from the selection of highly differentiated cell-lines as controls^14,16^.

In summary, the study shows that LCA has a strong, yet unselective effect on nephroblastoma and sarcoma cell-lines, slightly effecting highly differentiated cell-lines like podocytes. Further studies are needed to verify our results before dismissing LCA as an anti-cancer drug.

## Materials and methods

### Cell lines and cell culture

Nephroblastoma cell line WT CLS1 and sarcoma cell line SK NEP1, which was previously regarded as nephroblastoma were used^25^. HEK 293 is a well-known and commonly used embryonic kidney cell line used as a control. The expression markers and features of this cell line are well known and commonly used. Whereas RC 124 is a more specific kidney cell line with a closer proximity to the epithelial kidney layer. Both cells are none cancer cell lines. Furthermore, Human Podocytes as vulnerable and highly differentiated cells in kidneys were used as a control. These cells originate from mesenchymal metanephric blastema cells and therefore show most likely the same origin as nephroblastoma cells.

Cell line WT CLS1 was maintained in IMDM (Gibco, Life Technologies, Darmstadt, Germany), SK NEP1 and RC 124 in McCoy’s 5A (Genaxxon Bioscience, Ulm, Germany), with 2 mM L-Glutamine, HEK 293 in DMEM (Biochrom, Berlin, Germany). All media were supplemented with 10–20% Fetal Bovine Serum (Life Technologies, Darmstadt, Germany) and Penicillin/Streptomycin (10,000 U/ml/10,000 µg/ml, Biochrom, Darmstadt, Germany). All cells were cultivated at 37 °C, 5% CO_2_-athmosphere and a relative humidity of 95%.

Primary human podocytes were transfected with temperature sensitive SV40 and cultured as previously described^21^. Cells were used below passage 25. RPMI-medium was supplemented with 10% FBS (both Gibco, Life Technologies, Carlsbad, CA, USA) 100U/ml Penicillin, 0.1 mg/ml Streptomycin (Gibco), Glutamine, and Insulin, Transferrin, and Sodium Selenite Mixture (ITS) (Sigma). For flow cytometry cells were seeded on 6 well-plates (Sarstedt, Nümbrecht) and grown at 33 °C till 70–80% confluency. Cells were then differentiated at 38 °C for 10–14 days.

### Treatment with LCA

On day 1, the cells were seeded in the concentration according to the assay protocol. On day 2, the treatment with LCL took place in final concentrations of 0.1, 0.3, 1, 50, 100, 300, 400 μM.

### Chemical compounds, biological reagents and drugs

Lithocholic bile acid (LCA) (Sigma-Aldrich, Munich, Germany) was dissolved in 100% DMSO (Sigma-Aldrich, Munich, Germany), all working solutions were prepared by dilution in cell specific medium. For Western-Blotting primary antibodies against Cyclophilin-A (1:5000; Cell Signaling, Danvers, USA), NRF2 (ab31163, 1:800, abcam, Cambridge, United Kingdom) and TGR5 (NBP2-23669, 1:800, Novus Biologicals, Centennial, USA) were used.

### Cellular proliferation assay

Proliferation assays were performed with RealTime-Glo MT Cell Viability Assay (Promega, Madison, USA) according to manufacturer’s protocol. The RealTime-Glo MT Cell Viabilitiy Assay is commonly used to determine the number of viable cells in culture by measuring the reduction potential of cells and thus metabolism. Briefly, cells were plated into 96 well opaque-walled assay plates (Greiner Bio-One, Kremsmünster, Austria) at density of 1 × 10^5^ cells/well overnight. Next day cells were treated with LCA together with RealTime-GloTM reagent according to the manufacturer's protocol and cultured at 37 °C, 5% CO_2_-athmosphere and a relative humidity of 95% for 48 h. Measurement of luminescence was performed using a microplate reader (Flex Station® 3, Molecular Devices, San Jose, CA, USA).

### Detection of apoptosis by flow cytometry

Cells were seeded in cell culture medium in 24-well plates (Greiner Bio-One) overnight. Podocytes were seeded on six-well plates and grown at 33 °C, until they reached 70–80% confluency. Podocytes were differentiated for 10 days at 38 °C. All cells were treated with LCA for inidicated time, harvested using Accutase (Capricorne Scientific Ebsdorfergrund, Germany), washed twice with PBS (Gibco, Life Technologies, Darmstadt, Germany) and resuspend in Annexin V Binding buffer (10 mM Hepes, 140 mM NaCl and 0.25 mM CaCl_2_). Apoptosis was detected by Annexin V-FITC (556419; BP Pharmingen, Heidelberg, Germany) and Propidium-Iodine (PI) (Invitrogen, Darmstadt, Germany) staining. Analysis was performed with a flow cytometer (FACSCanto™ II, Becton, Dickinson, and Company, Franklin Lakes, NJ, USA). Data was analyzed using BD FACSDiva™ (Becton, Dickinson, and Company, Franklin Lakes, NJ, USA).

### Caspase 3 and 7 activity assay

Caspase activity was measured by using the Caspase-Glo 3/7 Assay (Promega, Madison, USA) according to manufacturer’s protocol. The assay provides a luminogenic caspase-3/7 substrate, which generating a luminescent signal after caspase cleavage. Briefly, 1.000 cells for each cell-line per well were seeded in a 96-well plate overnight. On the second day cells were treated with various final concentrations of LCA in a range from 0 to 400 µM for 1 h, 12 h, 24 h and 48 h. After indicated time Caspase-Glo 3/7 Reagent was added in 1:1 dilution and incubated for 1 h at room temperature. Measurement of luminescence was performed using a microplate reader (Flex Station® 3, Molecular Devices, San Jose, CA, USA).

### Protein extraction and Western blot analysis

Protein extraction and standard procedures for Western blotting was performed as described previously^16,19^. Briefly, cells were lysed using RIPA Buffer with cOmplete™ Mini Protease Inhibitor Cocktail (Merck KGaA, Darmstadt, Germany). Proteins from cells were separated by SDS-PAGE and transferred to PVDF membranes using Trans-Blot® Turbo™ system (Bio-Rad, Hercules, California, USA). Membranes were incubated with the indicated primary antibodies. Detection was performed with either horseradish peroxidase-labeled goat anti-mouse or anti-rabbit secondary antibodies (R&D systems, Minneapolis, United States) followed by enhanced chemiluminescence (SuperSignal™ West Atto Ultimate Sensitivity Substrat, Thermo Fisher Scientific, Waltham, Massachusetts, USA). Bands were visualized using Super Rx-N (Fujifilm, Tokio, Japan). Densitometric analysis was performed by ImageJ Software on the scanned blots, with proteins levels normalized to Cyclophilin-A.

### Statistics

All data were analyzed with Image Studio Lite (Version 5.2, Li-COR Biotechnology, Lincoln, United States), FlowJo (Version 7.6.2, BD Biosciences, San Jose, United States), ImageJ and GraphPad Prism (Version 9, GraphPad, San Diego, CA, USA). Differences between groups were calculated using two-way ANOVA with Dunnett’s multiple comparisons test. Data are presented as mean ± standard error of mean (SEM). The level of significance was set at *p* < 0.05.
